# Boreal Tintinnid Assemblage in the Northwest Pacific and Its Connection with the Japan Sea in Summer 2014

**DOI:** 10.1371/journal.pone.0153379

**Published:** 2016-04-07

**Authors:** Haibo Li, Zhiqiang Xu, Wuchang Zhang, Shaoqing Wang, Guangtao Zhang, Tian Xiao

**Affiliations:** 1 Key Laboratory of Marine Ecology and Environmental Sciences, Institute of Oceanology, Chinese Academy of Sciences, Qingdao, China; 2 Laboratory for Marine Ecology and Environmental Science, Qingdao National Laboratory for Marine Science and Technology, Qingdao, China; 3 The Marine Biological Museum of the Chinese Academy of Sciences, Institute of Oceanology, Chinese Academy of Sciences, Qingdao, China; 4 University of Chinese Academy of Sciences, Beijing, China; National Taiwan Ocean University, TAIWAN

## Abstract

Tintinnids are planktonic ciliates that play an important role in marine ecosystem. According to their distribution in the world oceans, tintinnid genera were divided into several biogeographical types such as boreal, warm water, austral and neritic. Therefore, the oceanic tintinnid assemblage could be correspondingly divided into boreal assemblage, warm water assemblage and austral assemblage. The purpose of this study was to investigate the characteristics of boreal tintinnid assemblage in the Northwest Pacific and the Arctic, and to identify the connection between boreal tintinnid assemblage and neighboring assemblages. Surface water samples were collected along a transect from the East China Sea to the Chukchi Sea in summer 2014. According to the presence of boreal genera and warm water genera, three tintinnid assemblages (the East China Sea neritic assemblage, the Japan Sea warm water assemblage, and the boreal assemblage) were identified along the transect. The boreal assemblage extended from the Chukchi Sea to the waters north of the Sōya Strait. Densities peaks occurred at stations in the two branches of the Alaska Current and decreased both northward and southward. The densities were <10 ind./dm^3^ at most stations in Arctic region. The dominant genera (*Acanthostomella*, *Codonellopsis*, *Parafavella*, and *Ptychocylis*) accounted for 79.07±29.67% (n = 49) of the abundance in the boreal assemblage. The densities of the dominant genera covaried with strongly significant positive correlations. Tintinnids with lorica oral diameter of 22–26 μm and 38–42 μm were dominant and contributed 67.35% and 15.13%, respectively, to the total abundance in the boreal assemblage. The distribution and densities of tintinnids in the study area suggest that the Sōya Strait might be a geographical barrier for tintinnids expansion.

## Introduction

Tintinnids are planktonic ciliates with shells (lorica). Taxonomically, tintinnids belong to the subclass Choreotrichia, class Spirotrichea [1]. As planktonic ciliates, tintinnids play an important role in the transfer of matter and energy between the microbial food web and the classic food chain in the marine planktonic ecosystem [2]. The morphology and size of the lorica have conventionally been used as taxonomic criteria despite plasticity of the lorica in some genera [3]. The lorica oral diameter (LOD; diameter of the mouth end of the lorica) is related to the size of food items: the size of the largest prey is about 45% of the LOD and the size of the preferred prey (removed at maximum rates) is about 25% of the LOD [4].

Tintinnid genera have been classified as cosmopolitan, neritic, warm water, boreal, and austral biogeographical types based on their distribution in the global ocean [5,6]. Correspondingly, according to the presence of each biogeographical genera type, there should be boreal, warm water and austral tintinnid assemblages in the oceanic water from Arctic to Antarctic. In the neritic waters are the neritic assemblages. There are also mixed assemblage at the edges of neighboring assemblages. Since there are only three oceanic assemblages extending from the Arctic to the Antarctic, each of the assemblage extends on a scale ≥3000 km (mega–scale) [7]. There are some studies about the austral assemblage and its mixing with warm water assemblage in the southwestern Atlantic Ocean [8–11], the warm water assemblage in Indian Ocean [12] and southeastern Pacific Ocean [13]. There are also some studies on tintinnid diversity at such large geographical scales in Mediterranean Sea [14–16]. In the Mediterranean Sea, both tintinnid species richness (10–25) and community averages of LOD (28–38 μm) increased from west to east. The dominant species were different in different areas, too [16]. Most tintinnid species showed a clear regional distribution and a well–defined tintinnid assemblage characterized each oceanic province along a transect from 42°N to 43°S across the Mediterranean Sea, the Red Sea, the Arabian Sea, the Indian Ocean and the Tasman Sea. For example, *Xystonella* and *Xystonellopsis* were considered as typical for the Mediterranean Sea. *Epiplocyloides reticulate* was considered as typical for the Red Sea [12].

Data on boreal tintinnid assemblages are scarce. There are few studies in the boreal assemblage from the northern Pacific [17] to Arctic [17,18]. Dolan et al. [18] found that *Ptychocylis urnula* or *Salpingella faurei* was the most abundant species in Arctic region. *Acanthostomella norvegica*, *Codonellopsis frigida*, *C*. *contracta*, *Parafavella subrotundata*, *P*. *ventricosa*, and *P*. *obtusa* were important components of tintinnid abundance in the oceanic waters, while *Ptychocylis* spp. and *Tintinnopsis* spp. were important in the shelf waters in Arctic and subarctic Pacific oceans [17]. These studies are limited in space coverage and low resolution in station numbers and distances between stations. In this paper, we studied tintinnid assemblages along a transect across the whole boreal assemblage from the Arctic to the eastern end of the Oyashio Current and south to the Japan Sea. The high resolution of station arrangement revealed the spatial variation of the assemblage. The aims of this study were (1) to describe the characteristics of the boreal tintinnid assemblage from 77.13°N in the Chukchi Sea to the northwest subarctic gyre in the Pacific, and (2) to investigate the connection between boreal tintinnid assemblage and that in the Japan Sea.

## Materials and Methods

**Fig 1:**
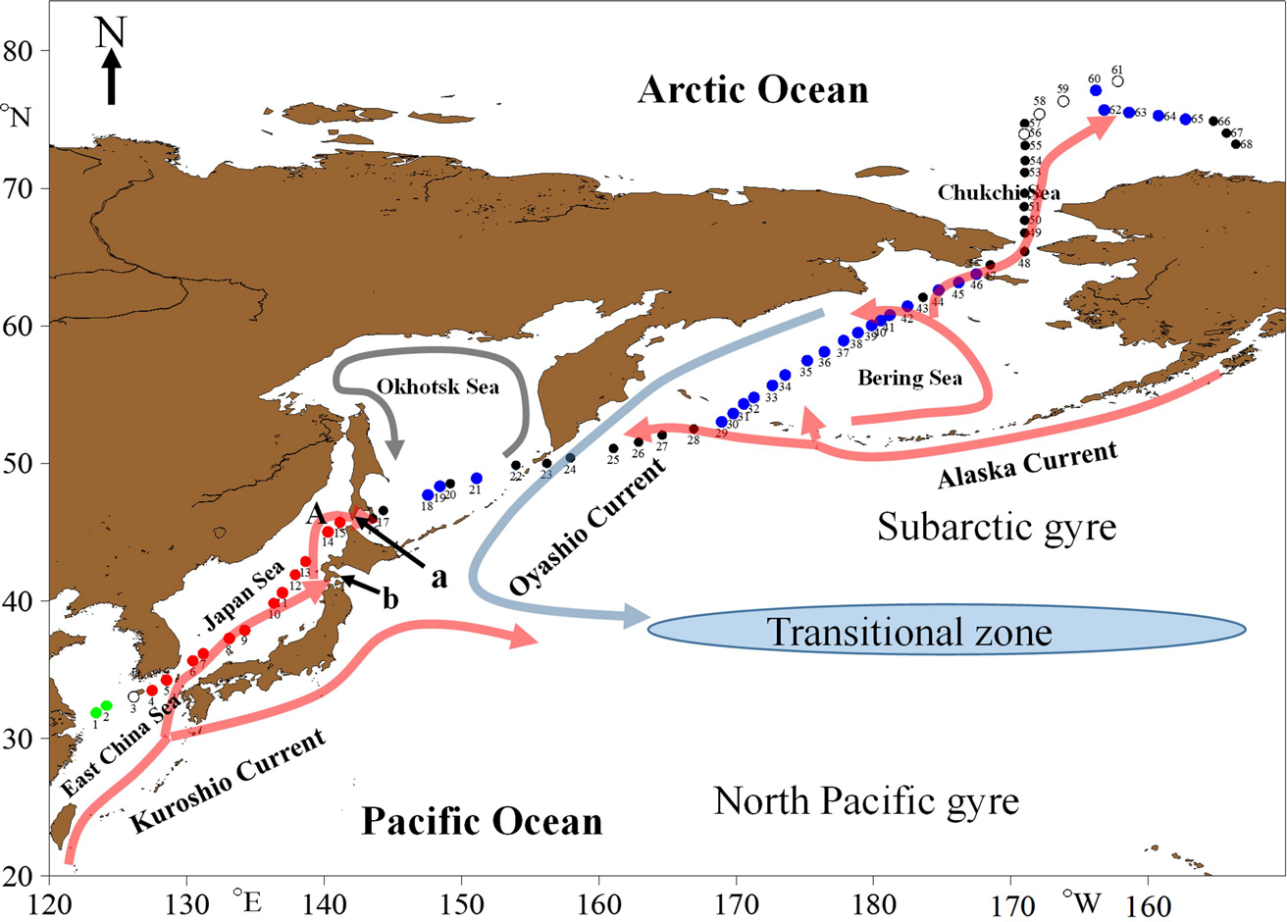
Locations of sampling stations and schematic of circulation in summer in the research area. Different colors of the stations indicate different tintinnid assemblages. Green filled circles: East China Sea neritic assemblage; red filled circles: Japan Sea warm water assemblage; blue filled circles: boreal assemblage without neritic genera influence (B1 assemblage); black filled circles: boreal assemblage with neritic genera influence (B2 assemblage); open circles: no tintinnid was found. A: Soya Current. a: Sōya Strait, b: Tsugaru Strait. Current positions are according to Springer et al. [19], Longhurst [20], Senjyu [21] and Steele et al. [22].

Tintinnids in surface waters were sampled during “The 6^th^ Chinese National Arctic Research Expedition” (11 July–31 August, 2014) on board R/V *XUELONG* along a transect (10290 km long) that started in the East China Sea, passed through the Japan Sea, Okhotsk Sea, and Bering Sea, and finished in the Chukchi Sea. There were 68 stations approximately evenly distributed along the transect, the distances between the adjoining stations were in the range of 60–480 km (on average 180±90 km, n = 67, Fig 1). No specific permissions were required to collect water samples because the stations were located in international waters. The field studies did not involve or impact on any endangered or protected species. The stations in the Japan Sea were in the path of the Tsushima Current, a branch of the Kuroshio Current, which influences the East China Sea and the Japan Sea, while the stations north of the Sōya Strait were influenced by two major North Pacific currents (the Oyashio Current and the Alaska Current) and currents flowing from the Bering Sea to the Chukchi Sea [19–22].

At each station, 80 dm^3^ of surface (5 m depth) seawater was collected using an underway sampling system. The water was then gently filtered through a small net (mesh pore size 10 μm). The samples in the cod end of the net (about150 cm^3^) were transferred into sample bottles and immediately fixed with Lugol’s solution (1% final concentration). The samples were placed at cool, dark environment for preservation. At the same time, the temperature and salinity of the surface seawater and chlorophyll *a* (Chl *a*) concentration were measured using a CTD sampling system (Seabird 21).

In the laboratory, one subsample (25 cm^3^ or a larger volume if tintinnids were scarce) from each concentrated sample was settled in an Utermöhl counting chamber for at least 24 h and examined using an Olympus IX 71 inverted microscope (100× or 400×). At least 20 individuals (if possible) of each species were photographed and measured. Tintinnid species were identified based on lorica morphology and size according to the literatures [23–25]. SPSS version 16 statistical software was used to perform a correlation analysis.

## Results

### Hydrography

**Fig 2:**
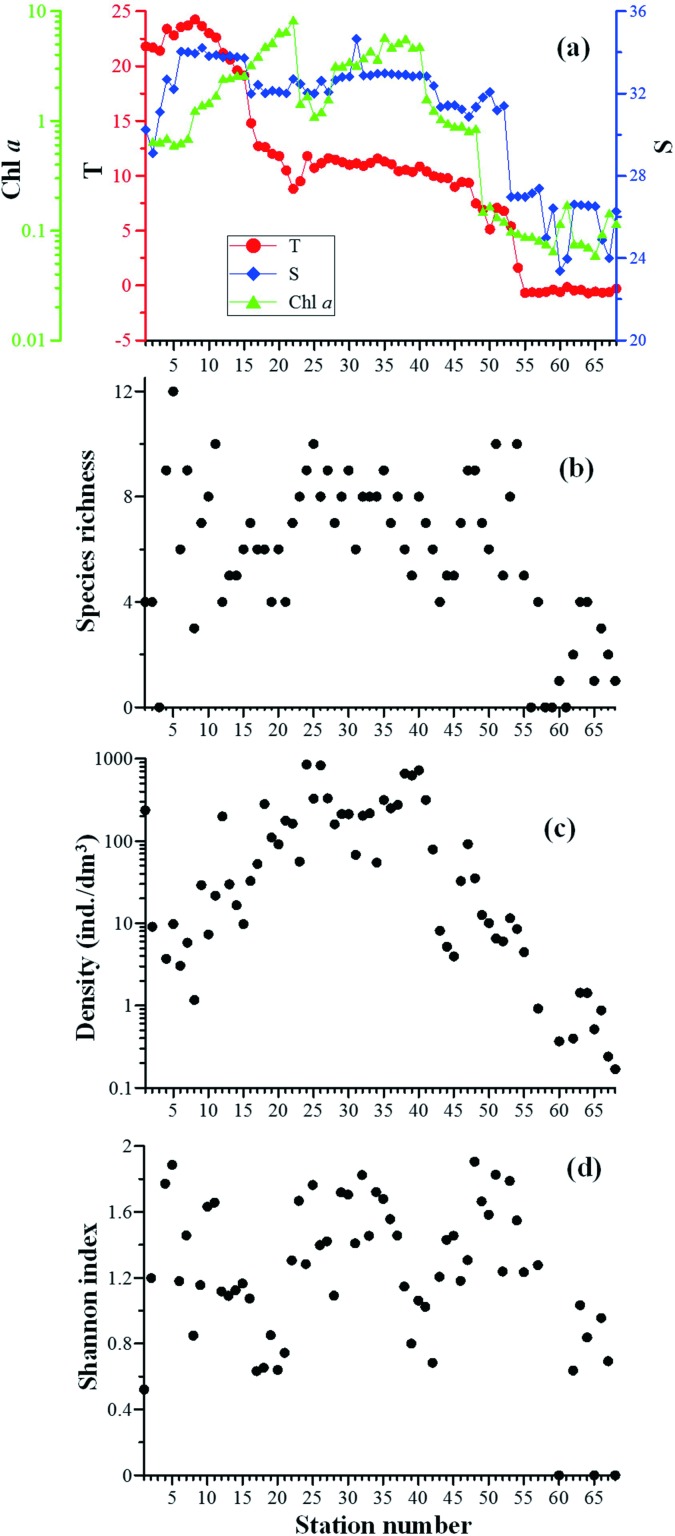
Distribution of temperature (T/°C), salinity (S) and Chl *a* (μg/dm^3^) (a), tintinnid density (b), species richness (c) and Shannon index (d) at each station.

**Fig 3:**
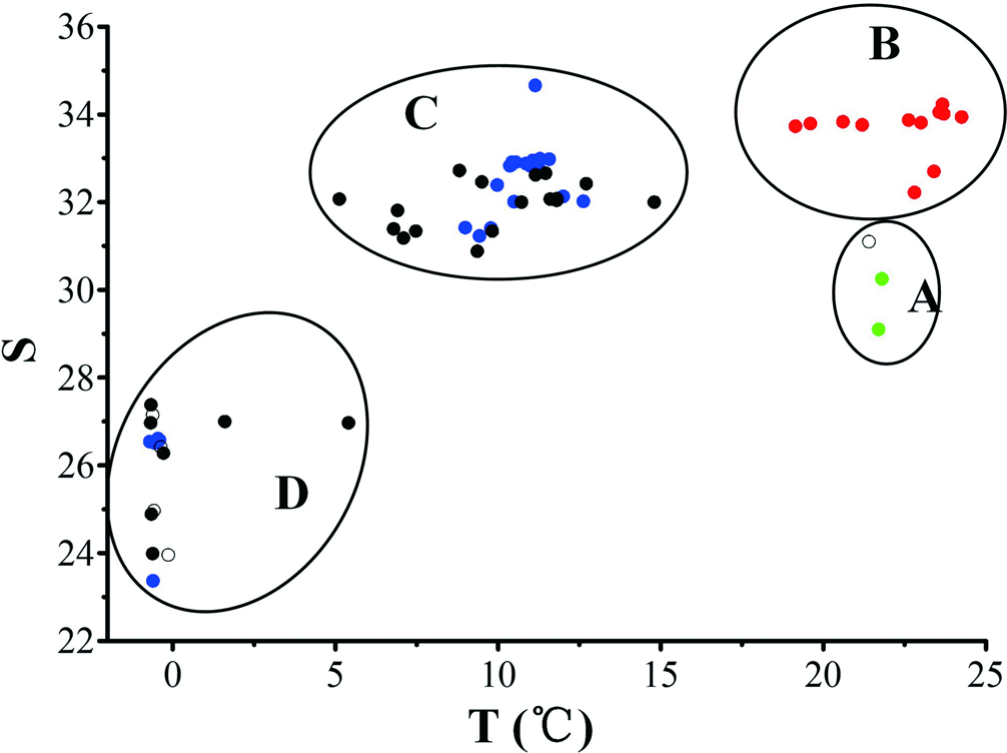
Temperature (T)-salinity (S) diagram and water mass division along the transect. A: East China Sea Water; B: Japan Sea Water; C: Subarctic Pacific Water; D: Coastal Arctic Water. Green filled circles: East China Sea assemblage; red filled circles: Japan Sea assemblage; blue filled circles: B1 assemblage; black filled circles: B2 assemblage; open circles: no tintinnid was found.

The surface water temperature ranged from –0.68°C (St. 55) to 24.25°C (St. 8). There were two sharp drops in temperature along the transect. The temperature increased slightly from St. 1 to St. 8, and then dropped rapidly from St. 9 (23.66°C) to St. 22 (10.49°C). It remained stable in the North Pacific and Bering Sea, then dropped rapidly from St. 47 (9.36°C) and remained below 0°C after St. 55 (Fig 2).The surface water salinity increased gradually from the East China Sea to the Japan Sea, and then remained at a high level (30.88–34.66) between the Japan Sea and the Bering Strait. The salinity dropped at St. 53 and remained at a low level (<27.38) in the Chukchi Sea (Fig 2). According to the T–S diagram, the water mass along the transect could be divided into East China Sea Water, Japan Sea Water, Subarctic Pacific Water and Coastal Arctic Water. St. 1–St. 3 located in East China Sea Water with high temperature but the salinity was relatively low. The stations between St. 4 and St. 15 located in the Japan Water with high temperature and high salinity. St. 53–St. 68 located in the Coastal Arctic Water with low temperature and low salinity (Fig 3).

The surface Chl *a* concentration ranged from 0.06 μg/dm^3^ (St. 65) to 8.32 μg/dm^3^ (St. 22). It increased gradually from the East China Sea to the northeastern edge of the Okhotsk Sea and reached a maximum at St. 22 before quickly falling to a relatively low level (<1.44 μg/dm^3^). Chl *a* concentration were high (3.17–5.80 μg/dm^3^) in the south part of the Bering Sea. Chl *a* concentration in the Chukchi Sea was very low (<0.2 μg/dm^3^; Fig 2).

### Species richness, densities of tintinnids and their relationship with environmental factors

**Fig 4:**
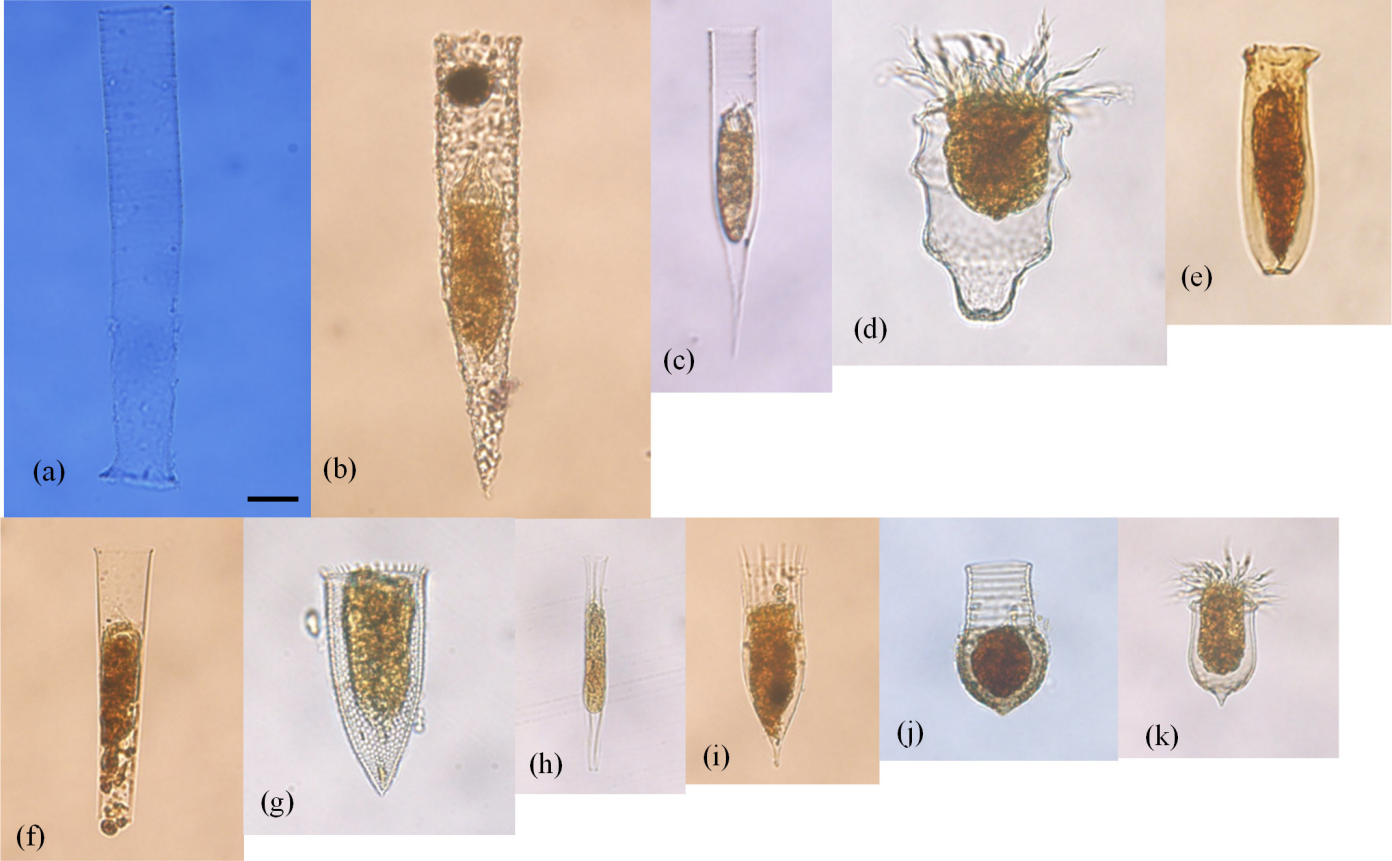
Photosof some tintinnid species occurred in this study. (a): *Leprotintinnus pellucidus*; (b): *Tintinnopsis radix*; (c): *Helicostomella subulata*; (d): *Ptychocylis obtusa*; (e): *Amphorides quadrilineata*; (f): *Eutintinnus stramentus*; (g): *Parafavella jorgenseni*; (h): *Salpingella faurei*; (i): *Dadayiella ganymedes*; (j): *Codonellopsis frigida*; (k): *Acanthostomella norvegica*. Scale bar: 20 μm.

**Fig 5:**
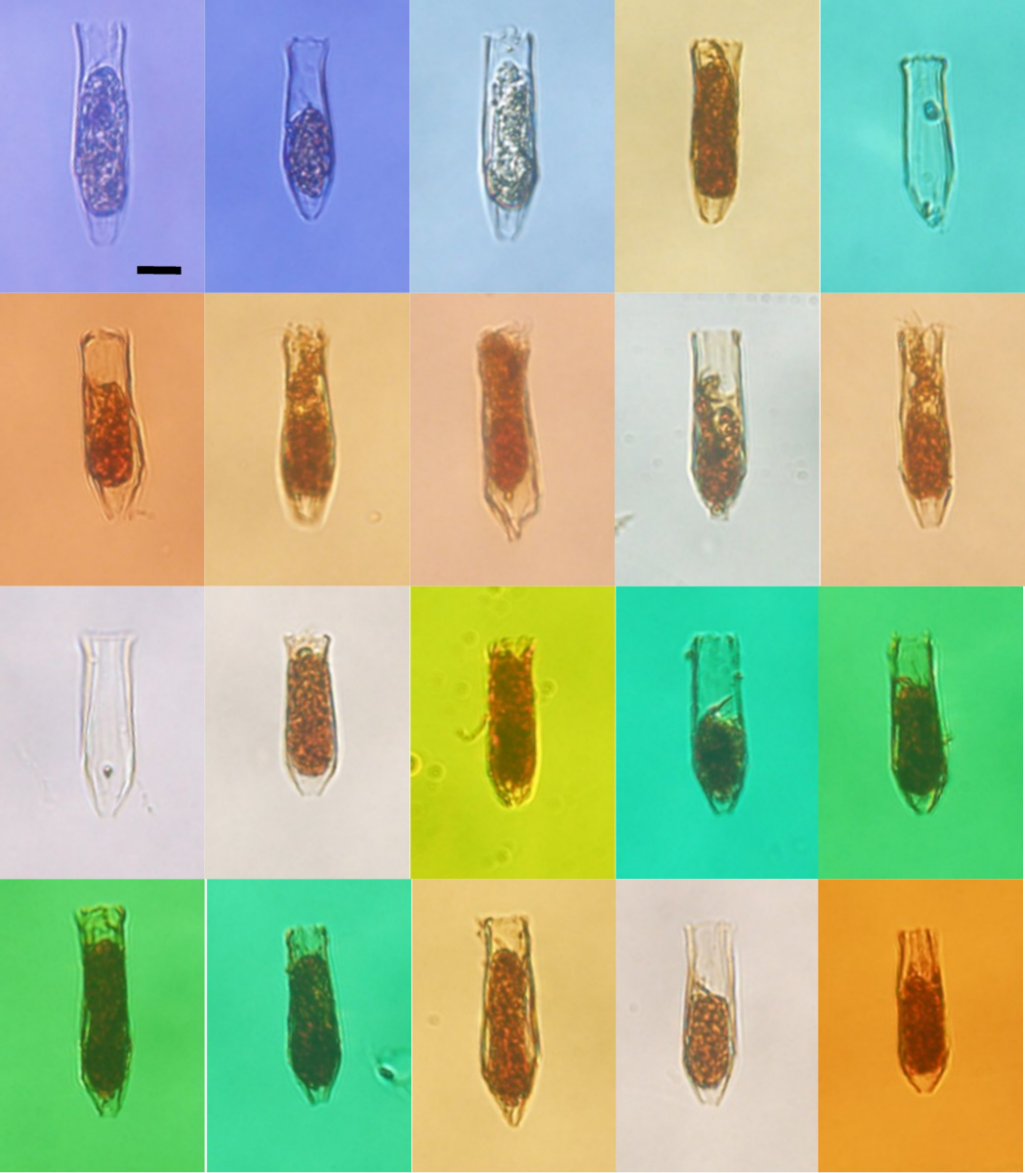
Photos of 20 different individuals of undetermined tintinnid. Scale bar: 10 μm.

In total, 54 tintinnid species from 21 genera were identified (Figs 4 and 5; Table 1). There was an undetermined species (Fig 5) with hyaline, cylindro–conical in shape, and open at both ends. The lorica length was about 44.88±3.97 μm, the LOD was 10.70±1.19 μm, and the aboral diameter was 3.62±0.77 μm (n = 20). We discussed it as a separate undetermined species and genus in this study. Thirty three species occurred at less than five stations and most (22) of them had low densities (<1 ind./dm^3^; Table 1).

**Table 1:** List of tintinnid species, the station number of occurrence (n) and maximum density (Max, ind./dm^3^).

Species	n	Max	Species	n	Max	Species	n	Max
**Boreal genera**			***Eutintinnu sapertus***	**2**	**2.05**	***T. baltica***	**4**	**0.93**
***Parafavella denticulata***	**9**	**32.08**	***E*. *fraknoii***	**3**	**1.46**	***T*. *beroidea***	**6**	**16.27**
***P*. *elegans***	**4**	**0.66**	***E*. *lusus–undae***	**8**	**3.08**	***T*. *japonica***	**2**	**0.53**
***P*. *faceta***	**26**	**53.47**	***E*. *mirabilis***	**2**	**0.27**	***T*. *kofoidi***	**1**	**0.13**
***P*. *gigantea***	**4**	**1.95**	***E*. *pacificus***	**6**	**2.83**	***T*. *mayeri***	**4**	**0.53**
***P*. *hadai***	**5**	**19.96**	***E*. *rectus***	**1**	**1.29**	***T*. *nana***	**1**	**15.80**
***P*. *jorgenseni***	**37**	**199.15**	***E*. *stramentus***	**7**	**27.94**	***T*. *radix***	**2**	**4.11**
***P*. *pacifica***	**5**	**79.26**	***Protorhabdonella striatura***	**1**	**0.26**	***T*. *rapa***	**3**	**1.68**
***P*. *promissa***	**4**	**0.66**	***Salpingella curta***	**1**	**1.30**	***T*. *tubulosoides***	**1**	**0.30**
***P*. *ventricosa***	**9**	**32.08**	***S*. *faurei***	**16**	**8.37**	***T*. *urnula***	**1**	**0.13**
***Ptychocylis obtusa***	**42**	**59.88**	***Steenstrupiella gracilis***	**1**	**0.13**	**Warm water genera**		
**Cosmopolitan genera**			***S*. *robusta***	**1**	**0.08**	***Ascampbelliella retusa***	**1**	**0.25**
***Acanthostomella conicoides***	**1**	**0.08**	***S*. *steenstrupii***	**3**	**0.66**	***Proplectella expolita***	**6**	**6.44**
***A*. *minutissima***	**2**	**1.03**	**Neritic genera**			***Rhabdonella cornucopia***	**3**	**0.40**
***A*. *norvegica***	**44**	**478.09**	***Leprotintinnus pellucidus***	**14**	**63.38**	***Rhabdonella* sp.**	**1**	**0.12**
***Amphorides brandti***	**1**	**1.11**	***Favella azorica***	**3**	**199.18**	**Ungrouped genera**		
***A*. *minor***	**1**	**0.25**	***F*. *panamensis***	**2**	**0.77**	***Climacocylis scalaroides***	**1**	**0.08**
***A*. *quadrilineata***	**13**	**55.95**	***Helicostomella subulata***	**7**	**51.29**	***Coxliella cymatiocoides***	**1**	**0.98**
***Codonellopsis frigida***	**33**	**45.16**	***Stenosemella nivalis***	**2**	**0.26**	**Undetermined**	**20**	**34.48**
***Dadayiella ganymedes***	**9**	**3.47**	***Tintinnopsis acuminata***	**11**	**15.80**			

Tintinnid densities ranged between 0 and 849.21 ind./dm^3^ (Fig 2). There were generally high densities between the Okhotsk Sea and southwestern Bering Sea, especially at stations St. 24–St. 27 and St. 35–St. 41. No tintinnid was found at St. 3, St. 56, St. 58, St. 59, and St. 61. Tintinnid densities were extremely low (<13 ind./dm^3^) in the Chukchi Sea (Fig 2).

The tintinnid species richness at individual stations ranged from 0 to 12, and was relatively high (3–12 species per station) in the northeastern East China Sea, the southwestern Japan Sea, and the northern Bering Sea. Tintinnid species richness declined after St. 55 (<4 species per station; Fig 2).

**Fig 6:**
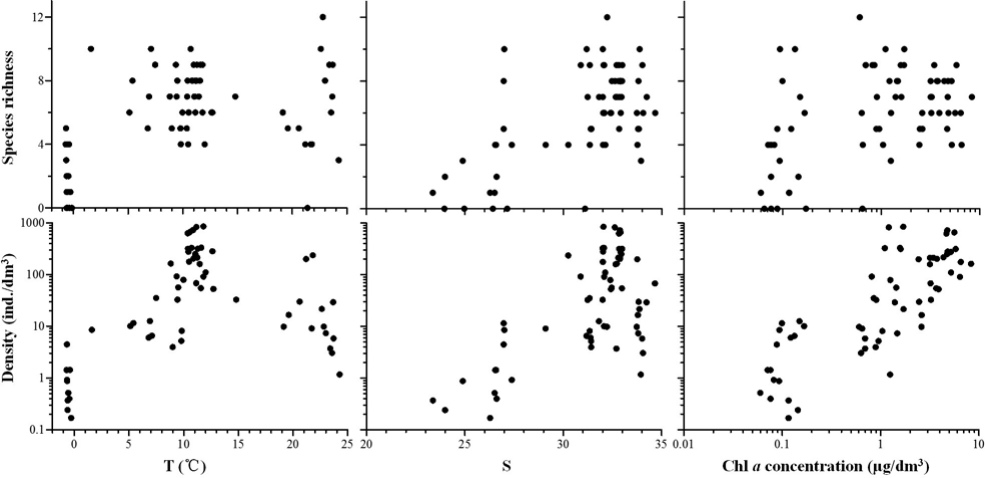
Responses of tintinnid species richness and density with the variation of temperature (T), salinity (S) and chlorophyll *a* (Chl *a*) concentration.

Correlation analysis identified significant positive correlations between tintinnid species richness and densities and temperature, salinity, and Chl *a* concentration (all *P*<0.01; Table 2). Tintinnid species richness and densities were very low when the temperature was <5°C, but were high when the temperature was between 8°C and 14°C. Tintinnid species richness and densities increased as salinity increased. Tintinnid species richness initially increased with an increase in Chl *a* concentration and then stabilized, while densities increased steadily as Chl *a* concentration increased (Fig 6).

**Table 2:** Spearman’s rank correlation coefficient between environmental and biological variables.

	T	S	Chl *a*
**Species richness**	0.362^**^	0.471^**^	0.373^**^
**Densities**	0.384^**^	0.534^**^	0.784^**^

T: temperature, S: salinity, Chl *a*: chlorophyll *a* concentration.

**: Correlation is significant at the 0.01 level (2–tailed).

### Distribution patterns of tintinnid genera

**Fig 7:**
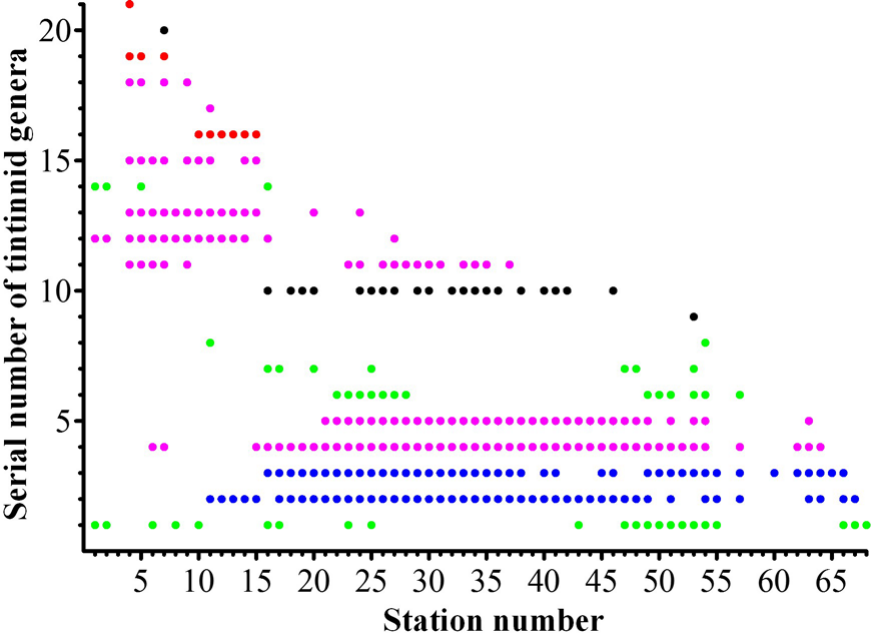
Occurrence of tintinnid genera at each station. Note that the undetermined species was listed as a genus. Blue filled circles: boreal genera; pink filled circles: cosmopolitan genera; green filled circles: neritic genera, red filled circles: warm water genera, black filled circles: ungrouped genera. (Serial number of tintinnid genera. 1: *Tintinnopsis*; 2: *Parafavella*; 3: *Ptychocylis*; 4: *Acanthostomella*; 5: *Codonellopsis*; 6: *Leprotintinnus*; 7: *Helicostomella*; 8: *Stenosemella*; 9: *Coxliella*; 10: undetermined; 11: *Salpingella*; 12: *Eutintinnus*; 13: *Amphorides*; 14: *Favella*; 15: *Dadayiella*; 16: *Proplectella*; 17: *Protorhabdonella*; 18: *Steenstrupiella*; 19: *Rhabdonella*; 20: *Climacocylis*; 21: *Ascampbelliella*).

**Fig 8:**
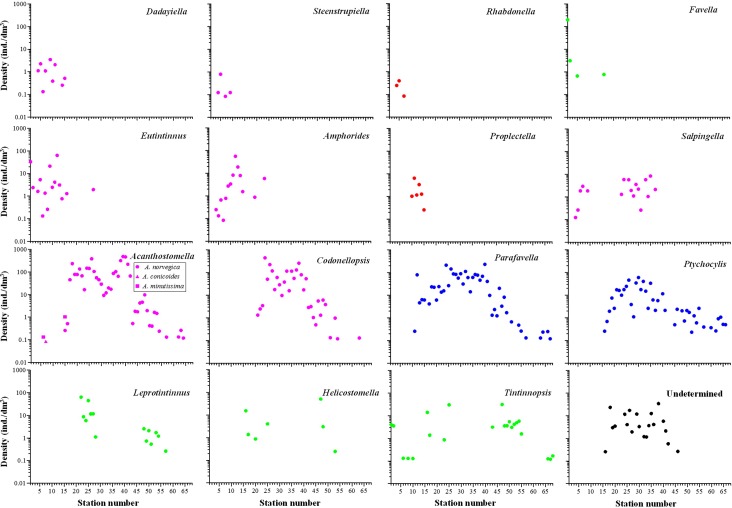
Density distribution of tintinnid genera which occurred at more than 2 stations. Blue filled circles: boreal genera; pink filled circles: cosmopolitan genera; green filled circles: neritic genera, red filled circles: warm water genera, black filled circles: ungrouped genera.

Among the 21 tintinnid genera, there were 2 boreal genera, 8 cosmopolitan genera, 4 neritic, and 3 warm water genera. Two genera and the undetermined species were not grouped into any biogeographical type (Table 1). The occurrence of each tintinnid genus by station was shown in Fig 7. Five genera (*Ascampbelliella*, *Climacocylis*, *Coxliella*, *Protorhabdonella*, and *Stenosemella*) were found at only one or two stations and their densities were <1 ind./dm^3^ (Table 1). Density distribution of other genera were shown in Fig 8.

*Proplectella* and *Rhabdonella* were warm water genera, *Proplectella* contained one species (*Proplectella expolita*) and *Rhabdonella* contained two species (*Rhabdonella cornucopia* and *Rhabdonella* sp.). They occurred at stations in the Japan Sea (Fig 8).

*Favela*, *Leprotintinnus*, *Helicostomella* and *Tintinnopsis* were neritic genera. *Favella* occurred at 4 stations in the Japan Sea and the East China Sea. *Leprotintinnus* and *Helicostomella* occurred at coastal stations east of the Kamchatka Peninsula and the Chukchi Sea. *Tintinnopsis* was found at coastal stations along the whole transect (Fig 8).

**Fig 9:**
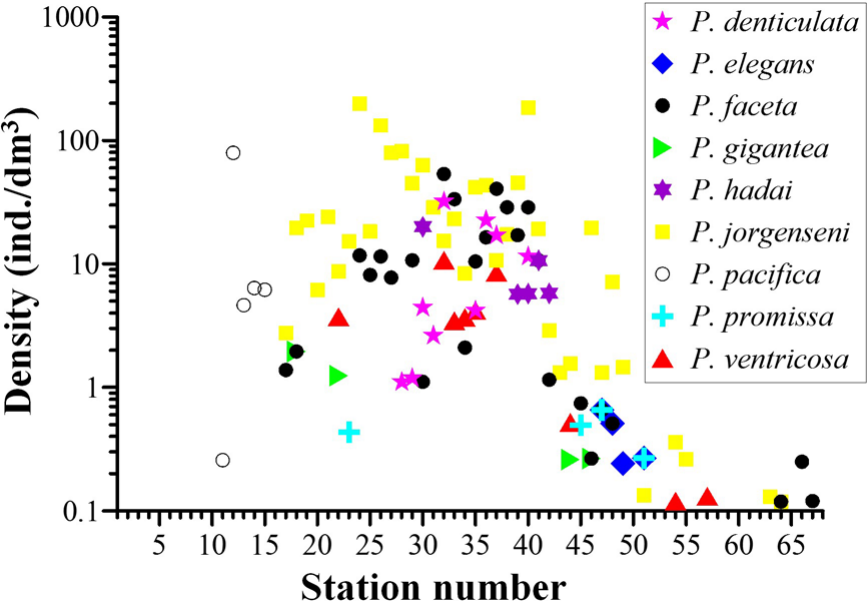
Density distribution of tintinnid species in genus *Parafavella*.

*Parafavella* and *Ptychocylis* belonged to boreal genera. *Ptychocylis obtusa* was the only species in genus *Ptychocylis*. It occurred north of the Sōya Strait (St. 16 –St. 66). Nine tintinnid species of the genus *Parafavella* were identified. The distribution of *Parafavella faceta* and *P*. *jorgenseni* was similar. Both species were first detected at St. 17. Their densities initially increased along the transect, then decreased in the Arctic. *P*. *elegans* occurred in the Bering Strait with low densities of <0.66 ind./dm^3^ (Table 1). *P*. *denticulata* occurred between St. 28 and St. 40 where most tintinnid species of the genus *Parafavella* were highly abundant. Most species in *Parafavella* only occurred at stations north of the Sōya Strait with the exception that *P*. *pacifica* was only found in the northern Japan Sea (Fig 9).

The cosmopolitan genera Acanthostomella, Amphorides, Codonellopsis, Dadayiella, Eutintinnus, Salpingella, and Steenstrupiella contained 3, 3, 1, 1, 7, 2, and 3 species, respectively. Dadayiella ganymedes and Eutintinnus stramentus were identified in the Japan Sea but were not found north of the Sōya Strait. Codonellopsis frigida distributed at stations between St. 20 and St. 63. Acanthostomella norvegica mainly distributed north of the Sōya Strait. It also occurred at St. 15 in the Japan Sea with extremely low density (0.26 ind./dm^3^). The genus Salpingella distributed in the southern (St. 4–St. 9) and middle (St. 23–St. 37) sections of the transect (Fig 8).

### Tintinnid assemblages division

We used the occurrence of boreal genera and warm water genera as objective criteria of tintinnid assemblage division. The 5 stations with zero density (St. 3, St. 56, St. 58, St. 59, and St. 61) were excluded of assemblage division. The stations with both boreal and warm water genera were set according to T–S water mass division.

Neither warm water genera nor boreal genera tintinnid was identified at the two stations (St. 1 and St. 2) in the East China Sea. We considered the tintinnid assemblage at these two stations as East China Sea neritic assemblage. Warm water genera occurred in the Japan Sea (St. 4 to St. 15). At the stations between St. 11 and St. 15, warm water genera and *P*. *pacifica* belonging to boreal genera mixed. In the T–S diagram, these stations belong to the Japan Sea Water with high temperature (Fig 3). Therefore the tintinnid assemblage at St. 4 to St. 15 was considered as Japan Sea warm water assemblage. Tintinnid assemblage at stations north of St. 16 was considered as boreal assemblage with boreal genera occurred.

### Characteristics of boreal assemblage

**Fig 10:**
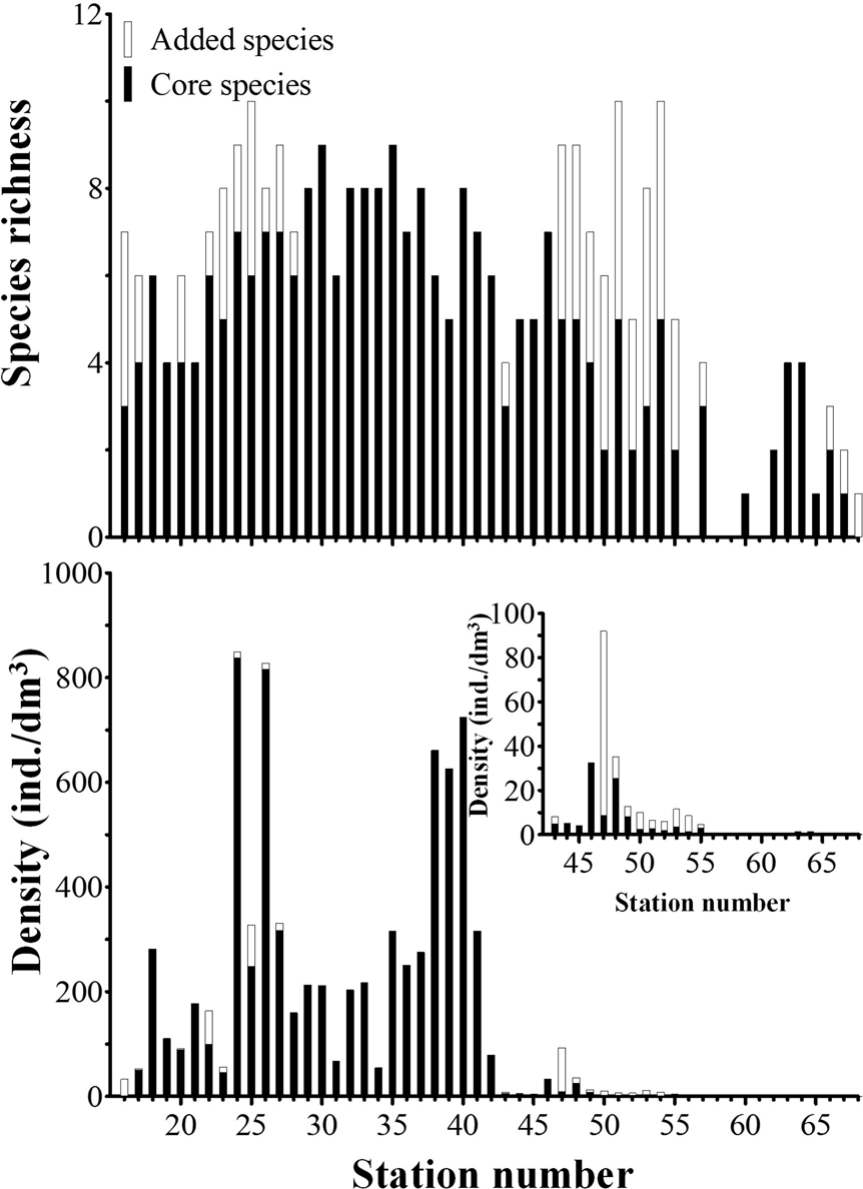
Species richness and density at stations in boreal assemblage.

The boreal assemblage covered the Subarctic Pacific Water and Coastal Arctic Water and has a difference in densities in the two waters. Total densities decreased sharply at stations in the Coastal Arctic Water and was <10 ind./dm^3^ at most stations north of the St. 49 (Figs 2 and 10).

**Fig 11:**
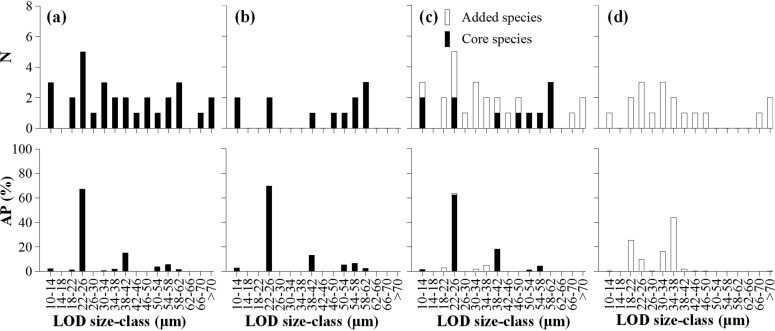
Number of species (N) and abundance proportion (AP) in each LOD size-class in boreal, B1, and B2 assemblages. Column (a): boreal assemblage; Column (b): B1 assemblage; Column (c): B2 assemblage; (d): added species in B2 assemblage.

There were 30 species with 14 LOD size–classes in the boreal assemblage. Species in LOD of 22–26 μm (*A*. *norvegica*, *C*. *frigida*, *Stenosemella nivalis*, *Tintinnopsis beroidea*, and *T*. *rapa*) accounted for 67.35% of the total abundance. The second LOD size-class with high contribution to abundance (15.13%) was 38–42 μm (*Amphorides quadrilineata* and *P*. *jorgenseni*). The other 23 species in LOD size–classes 10–14 μm, 18–22 μm, 26–30 μm, 30–34 μm, 34–38 μm, 42–46 μm, 46–50 μm, 50–54 μm, 54–58 μm, 58–62 μm, 66–70 μm, and >70 μm accounted for 17.52% of boreal assemblage abundance (Fig 11).

**Fig 12:**
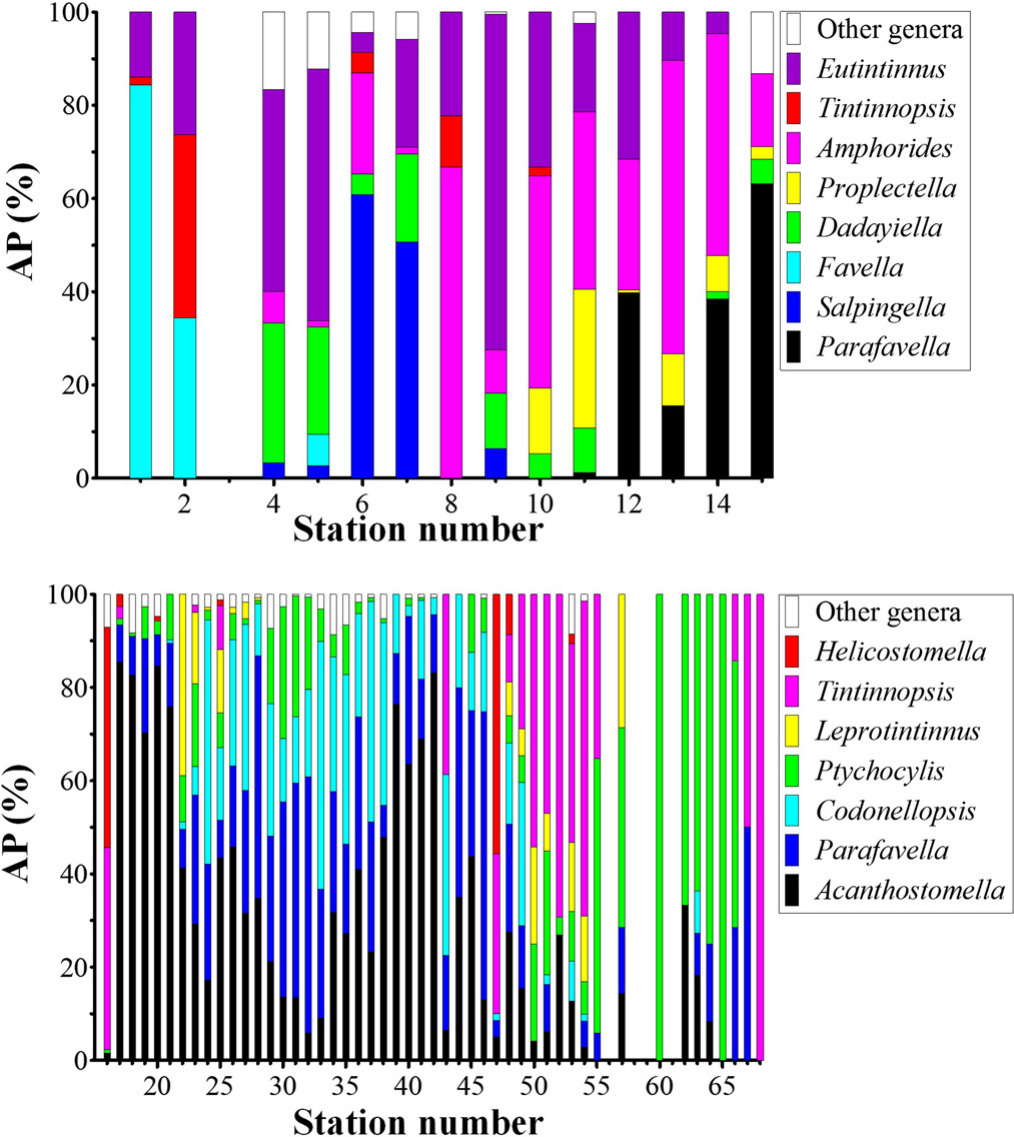
Abundance proportion (AP) of abundant genera at each station.

**Fig 13:**
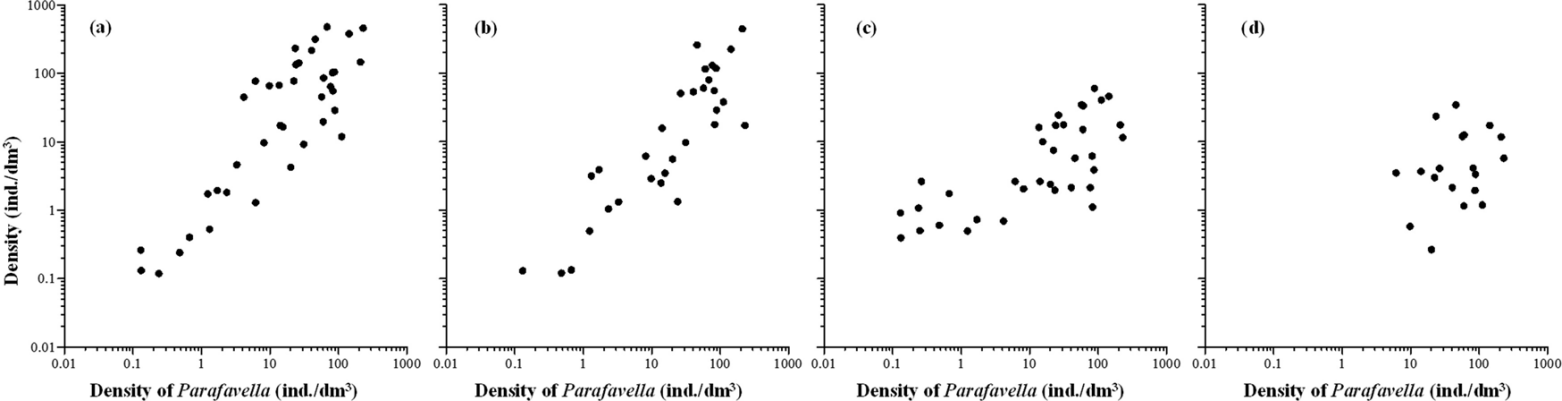
Relationship between densities of genus *Parafavella* and *Acanthostomella* (a); *Codonellopsis* (b); *Ptychocylis* (c); and undetermined tintinnid (d).

With maximum densities of 478.09, 445.16, 229.87, and 59.88 ind./dm^3^, respectively, the genera *Acanthostomella*, *Codonellopsis*, *Parafavella*, *Ptychocylis*, had higher maximum densities than other genera. On average, they contributed 79.07±29.67% (n = 49) to the abundance in the boreal assemblage (Fig 12). They also had consecutive distributions. The distribution patterns of *Acanthostomella*, *Codonellopsis*, *Parafavella*, *Ptychocylis*, and the undetermined species were similar. They centered in the Bering Sea with extensions both southward and northward. Their high densities centered in the Bering Sea and north of the Okhotsk Sea (St .18–St. 41) and decreased both southward and northward. The undetermined species occurred between St. 16 and St. 47 with densities of 0.27–23.43 ind./dm^3^ (Fig 8). The correlations among densities of them were strongly significant positive (all *P*<0.01; Table 3; Fig 13).

**Table 3:** Spearman’s rank correlation coefficient among density distribution of genera *Parafavella*, *Acanthostomella*, *Codonellopsis*, *Ptychocylis* and the undetermined species.

	Acanthostomella	Codonellopsis	Parafavella	Ptychocylis	Undetermined species
***Acanthostomella***	**1**				
***Codonellopsis***	0.786^**^	**1**			
***Parafavella***	0.799^**^	0.798^**^	**1**		
***Ptychocylis***	0.720^**^	0.677^**^	0.689^**^	**1**	
**Undetermined species**	0.698^**^	0.573^**^	0.641^**^	0.651^**^	**1**

**: Correlation is significant at the 0.01 level (2–tailed).

According to whether neritic genera tintinnid occurred, the boreal assemblage could be divided into two parts: boreal assemblage without neritic genera (B1 assemblage) and boreal assemblage with neritic genera (B2 assemblage). There were 12 species with 7 LOD size–classes in B1 assemblage, and these species were considered as core boreal species. In the B1 assemblage, two species (*A*. *norvegica* and *C*. *frigida*) with a LOD of 22–26 μm accounted for 69.65% (49.16% and 20.49%, respectively) of the total abundance. The second highest contributor to abundance (13.16%) was *P*. *jorgenseni* with a LOD of 38–42 μm (Fig 11). Core boreal species had high species richness at stations between St. 29 and St. 42, and decreased northward and southward, and extremely low in Arctic region (Fig 10).

Twenty nine species with 14 LOD size–classes were found in B2 assemblage. Among them, 11 species were core boreal species. Eighteen other species (added species) occurred in B2 assemblage, including 12, 4, and 1 species belong to neritic, cosmopolitan, and boreal genera, respectively.

Though the number of species and LOD size–classes increased in the B2 assemblage, the most abundant species in B2 assemblage were similar with those in B1 assemblage: species in LOD of 22–26 μm and 38–42 μm contributed to 63.74% and 18.22% to total abundance, respectively (Fig 11). The added species increased species richness in B2 assemblage, especially at stations in the Coastal Arctic Water. However, compared with core boreal species, densities of the added species were low and made up a small portion of total abundance at most stations in B2 assemblage. High portion mainly occurred at stations in the Arctic water where the total densities were low (Fig 10). If the core species were excluded, the added species with LOD size–classes of 34–38 μm, 18–22 μm, and 30–34 μm were dominant and accounted for 43.88%, 25.51%, and 16.51% of abundance in B2 assemblage, respectively (Fig 11).

## Discussion

### Hydrography and assemblages division

The division of tintinnid assemblage corresponded well with water mass division by T–S diagram. The East China Sea neritic assemblage and the Japan Sea warm water assemblage located in the East China Sea and the Japan Sea, respectively. The boreal assemblage was in the waters north of the Sōya Strait where the T–S diagram identified two water masses (Subarctic Pacific Water and Coastal Arctic Water).

The transect in this study passed through the following biological provinces: the North Pacific Epicontinental Sea Province (the Bering Sea and the Okhotsk Sea), the Pacific Subarctic Gyre Province, the Kuroshio Current Province (the Japan Sea), and the China Sea Coastal Province [20,26]. Our division of the East China Sea neritic assemblage and the Japan Sea warm water assemblage corresponded with the China Sea Coastal Province and the Kuroshio Current Province, respectively. The boreal assemblage in this research covered the North Pacific Epicontinental Sea Province and the Pacific Subarctic Gyre Province [20,26]. Our result was in consistent with Priede [26] who described the region north of the westerly current in the Northern Hemisphereas the polar (or boreal) biome. In the present study we used the term boreal to describe the assemblages in this biome.

The Japan Sea warm water assemblage was at the brim of the warm water assemblage. The East China Sea neritic assemblage only had 2 stations. Therefore these two assemblages in our study were not typical. The transect covered the boreal assemblage from its north end to south end. Our research is the first study of the boreal assemblage in a transect across the assemblage.

### Characteristics of the boreal assemblage

There were very few previous studies of tintinnids in the Arctic [17,18] and subarctic Pacific [17] regions. The study in subarctic Pacific [17] region only gave total densities and species names of dominant species in 15 stations.

The densities of tintinnids in the boreal assemblage had a sharp decrease from the Subarctic Pacific Water to Coastal Arctic Water (Figs 2 and 10). This decrease in densities were consistent with findings of previous studies [17,18]. Meanwhile, all tintinnid species occurred in the Chukchi Sea had been reported at other areas other than Arctic [17,18]. Therefore tintinnids might intrude into the Chukchi Sea through the Bering Strait by the help of current from the Bering Sea [19]. But most tintinnids cannot adapt to the extreme environment in the Chukchi Sea, this caused the low densities and tintinnid species richness in the Arctic region [17]. This also proved that there was no indigenous tintinnid species in the Arctic Ocean [17]. This is different from the case in Antarctic. Some species in genera *Cymatocylis*, *laackmanniella*, and *codonellopsis* only distributed in Antarctic region south of 58°S, and were considered as Antarctic indigenous tintinnids [10].

The positions of the high densities (St. 24–St.27, St. 35–St. 41) were corresponding with the two branches of the Alaska Current located in the Bering Sea. But we did not have current data, we were not sure whether the high densities were related with the current branches at the time of sampling. Previous study showed that tintinnid destiny was high in the area where water masses mixed [12]. Therefore we thought the high densities were caused by the mixing of branches of the Alaska Current and the Oyashio Current.

It was reported that *Ptychocylis urnula* or *Salpingella faurei* was the most abundant species in Arctic region [18], while the species in genera *Acanthostomella*, *Codonellopsis*, *Ptychocylis*, *Parafavella*, and *Tintinnopsis* were the most abundant in subarctic Pacific [17]. The similar abundant genera were found in our study with *Acanthostomella*, *Codonellopsis*, *Ptychocylis*, and *Parafavella* accounted for 79.07±29.67% (n = 49) of abundance at stations in the boreal assemblage (Fig 12).

One undetermined species that has not been reported in previous study was found in this study. According to its lorica characteristics, it may belong to the genus *Salpingella*. But it was different with *S*. *acuminata* or *S*. *faurei* that had been reported in the Arctic [18], and subarctic regions [17].

The distribution patterns of *Acanthostomella*, *Codonellopsis*, *Parafavella*, *Ptychocylis*, and the undetermined species were similar (Fig 13). Those genera was similar to “species group” proposed by Fager and Mcgowan [27]. Their positive correlation might be because that they had similar reactions to properties of the environment.

LOD is a valuable characteristic of tintinnid feeding activity. The maximal prey size ingested is about 45% of the LOD, while the preferred prey size is about 25% of the LOD [4]. Species in two LOD size–classes (22–26 and 38–42 μm) contributed 82.81% to the total abundance in B1 assemblage (Fig 11). This means that the prey size was limited to a small number of size–classes. Species in those two LOD size–classes were still the main contributors to total abundance in B2 assemblage, but the number of LOD size–classes increased. This indicated that the added species in B2 assemblage had different prey size–classes with the core species in boreal assemblage.

Boreal assemblage in our study was at the downstream of the Alaska Current, the characteristics of the assemblage in this transect should also apply to the area in the east part of the Alaska Current and to the entire Bering Sea.

### Obstruction of the Sōya Strait to tintinnid expansion

The Sōya Strait is about 40 km long and 20–40 m deep. The boreal assemblage and the Japan Sea warm water assemblage were separated by the Sōya Strait, which might be a natural geographic barrier for tintinnid species. Species including *Dadayiella ganymedes*, *Eutintinnus stramentus*, and *Proplectella expolita* were identified in the Japan Sea but were not found north of the Sōya Strait. *Acanthostomella norvegica* and *Codonellopsis frigida* mainly distributed north of the Sōya Strait. *S*. *faurei* was reported to be the most abundant species in the Arctic region [18]. In this study, it was identified in areas south of the Japan Sea and in the Northwest Pacific, but were not found in the northern Japan Sea or the Okhotsk Sea. We did not know the connection of the two distribution areas.

Lorica morphology of some tintinnid species might vary following the environmental change, especially species in genus *Parafavella* [28]. The variation of tintinnid lorica morphology made species classification inaccurate just based on lorica morphology. In our study, *Parafavella pacifica* was the only species in genus *Parafavella* occurred in the Japan Sea warm water assemblage. It may be an intraspecific variation of the species north of the Sōya Strait. The lorica shape changed to adapt to the Japan Sea environment.

As the Sōya Strait is a natural geographic barrier, it was not possible to observe mixing between warm water and boreal tintinnid assemblages where the Oyashio and Kuroshio Currents meet. There are limited biological data available for this transitional zone created by the mixing of the Oyashio and Kuroshio Currents [29]. *D*. *ganymedes* was distributed the farthest north from the East China Sea into the Japan Sea, whereas *Steenstrupiella* spp. were confined to the south. This pattern was consistent with the intrusion of the Kuroshio Current into the East China Sea [30]. Therefore, we suggest that *D*. *ganymedes* is the most likely species to exist in the region east of Japan where the Kuroshio and Oyashio Currents meet, whereas *Steenstrupiella* spp. are likely to be the first to disappear.

## Conclusions

With large space coverage and high resolution in station distribution, we identified three tintinnid assemblages (East China Sea neritic, Japan Sea warm water, and boreal assemblages) along the study transect. The tintinnid assemblage north of the Sōya Strait was boreal assemblage. There were peaks in densities at stations approximately between the two branches of the Alaska Current. The densities decreased both northward and southward. In Arctic region, the densities were extremely low (<10 ind./dm^3^ at most stations). *Acanthostomella*, *Codonellopsis*, *Parafavella*, and *Ptychocylis* were the dominant genera in this boreal assemblage. There were strong positive correlations between the densities of these four genera. In coastal waters, more species occurred and made the boreal assemblage much more complex both in tintinnid composition and LOD size–classes. The Sōya Strait forms a physical barrier between the boreal assemblage and the Japan Sea warm water assemblage.

## Supporting Information

S1 File All original data. (XLS)
